# Clinical Significance of Langerhans Cells in Squamous Cell Carcinoma of the Larynx

**DOI:** 10.1155/2012/753296

**Published:** 2012-02-09

**Authors:** Francisco Esteban, Francisco Ruiz-Cabello, Miguel Angel Gonzalez-Moles, Miguel Angel Lopez-Gonzalez, Rafael Funez, Maximino Redondo

**Affiliations:** ^1^Servicio de Otorrinolaringologia, Hospital Universitario Virgen del Rocío, Sevilla 41013, Spain; ^2^Servicio de Análisis Clínicos e Inmunología, Hospital Universitario Virgen de las Nieves, Granada 18014, Spain; ^3^Departamento de Medicina Bucal, Universidad de Granada, Granada 18001, Spain; ^4^Servicio de Anatomía Patológica, Hospital Costa del Sol, Marbella 29600, Málaga, Spain; ^5^Área de Laboratorios Clínicos, Hospital Costa del Sol, Universidad de Málaga, Carretera de Cádiz Km 187 29600, Marbella, Málaga, Spain

## Abstract

Langerhans cells (LCs) may be involved in the immunosurveillance against tumors as antigen-presenting cells. Our objective has been to determine the relevance of LC in progression of larynx squamous cell carcinomas and their relationship with different subpopulations of tumor-infiltrating cells. LCs were investigated by immunohistochemical methods using anti-CD1 antibody. LCs were detected in most of the primary tumors studied (44 out of 50) and also in metastases (6 out of 10) and recurrences (2 out of 3), but we did not find any statistical association between number of LCs and clinical-pathological parameters or survival. However, the number of LCs was increased in patients with evident infiltration of lymphocytes, mainly cytotoxic T cells. We can conclude that although LCs did not show clinical utility as prognostic marker, they may play a role in releasing an active immune response in larynx carcinomas, according to their ability to present antigens to sensitized T cells.

## 1. Introduction

Laryngeal squamous cell carcinomas display a limited and staged tendency to metastasize. This behaviour raises the question of whether local mechanisms exist inside the larynx which play a protective role by recognizing and inhibiting the spread of neoplastic cells. It is well known that T lymphocytes are the major cells involved in tumor cell kill, although T cells need to be activated by antigen presentation. One of the antigen-presenting cells is the Langerhans cells that constitute a small subpopulation (3–8%) of epidermal cells with dendritic processes. In addition, LCs have receptors for the Fc portion of IgG and the complement component C3b. Langerhans cells are characterized by two types of markers: an ultrastructural marker, the Birbeck granule, and different membrane markers: HLA-DR antigens, S-100 protein and CD1a.

Antigen-presenting Langerhans cells are now considered a population of cells of bone marrow origin [1], which pick up antigens encountered in the squamous epithelia of larynx and to migrate subsequently to the draining lymph nodes. Upon arrival in the paracortex of lymph nodes, the antigen-laden LCs transform into interdigitating cells and they present antigen to naive T lymphocytes. In addition LCs have the capacity to present complex protein antigens such as purified protein derivative of tuberculin and herpes simplex virus antigen to lymphocytes [1]. Therefore LCs may be involved in immunosurveillance against neoantigens associated with malignant transformation by transporting antigens to local lymph and presenting them to specific T cells. Furthermore, there is an increasing body of evidence supporting the hypothesis that LCs have a role in the immunosurveillance against a number of different tumors, such as nasopharyngeal carcinoma, gastric carcinoma, or papillary thyroid carcinoma [2–4]. 

A relationship between LCs and prognosis has been published in a number of different tumors [5, 6] raising the question of whether LCs have a role in the immunosurveillance against cancer. However, there are surprisingly few reports investigating the presence and significance of LCs in laryngeal tumors [7–9], one of the commonest cancers in the Mediterranean area. Our study is the first report of the association between LCs and the different subpopulations of tumor-infiltrating leukocytes in squamous cell carcinomas of the larynx. For this purpose we used a panel of monoclonal antibodies (Moabs) and immunohistochemistry techniques. LCs were detected in most of the tumors studied and also in metastases or recurrences. The presence of these cells in the metastases is another evidence of their extraepidermal origin and their relationship with antitumoral mechanisms. The number of LCs present only yielded significance in relation to other leukocyte subpopulations, emphasizing that LCs can serve as an indicator of defence of the host against the larynx carcinoma. The function of LCs in tumor immunobiology is discussed.

## 2. Material and Methods

### 2.1. Patients

All the patients were treated according to a relatively uniform philosophy during the study period. Surgery consisted predominantly of total laryngectomy and horizontal supraglottic laryngectomy in selected cases. Neck dissection was carried out in clinical stage IV disease and in stage III disease as indicated by the presence of cervical metastases and the specific site of the primary tumor. Twenty-seven tumors (54%) were classified as supraglottic, 14 glottic (28%), 2 (4%) subglottic, 4 (8%) transglottic and 3 (6%) were pyriform sinus carcinomas. All were male and none of the patients had received radiotherapy and/or chemotherapy prior to surgery. The youngest patient was 44 years old and the oldest 75 years old (mean age, 58 years). All were male. Diagnosis was confirmed in paraffin sections. Tumors were classified into 3 grades—Broders, modification by WHO (World Health Organization)—and all were scored according to Glanz's [10] and Jakobsson's grading for squamous cell cancer [11]. Cases were also carefully staged to conform to the 1995 criteria of the American Joint Committee for Cancer Staging and End Results Reporting. T-stage was recorded as follows: 4 (8%) T1, 4 (8%) T2, 30 (60%) T3 and 12 (24%) T4. N-stage: 34 N0 (68%), 4 N1 (8%), 5 N2 (10%), 7 N3 (14%), and in two cases neck exploration was not available in the clinical notes. The histological analyses were performed without any knowledge of the clinical stage, treatment, or the further course of the disease. Longest followup ranged between 96 and 144 months. Patients gave informed consent which was noted in the clinical history. All procedures followed the ethical standards of the hospital committee on human experimentation and the Helsinki Declaration (1975, 1983).

### 2.2. Monoclonal Antibodies 

The following Moabs were used: GRB1 against DR antigen; GRT2, which recognizes the common leukocyte antigen CD45; Leu 4, Leu 3a, Leu 2a, against CD3, CD4, CD8, respectively, (Becton Dickinson), Rutherford, NJ); Bear-1 against CD11b; OKT6 against CD1 (Ortho, Raritan, NJ); IOM-1 against CD20 (Immunotech, Marseille, France).

### 2.3. Immunohistochemical Analysis

Binding of Moabs to frozen sections was assayed by the techniques of alkaline immunophosphatase and streptavidin-biotin. Details of the methods and sample processing have been published elsewhere [12, 13]. Sections were examined with an Olympus BH-2 microscope. The intensity, composition and distribution of the inflammatory infiltrate were examined both qualitatively and quantitatively in all sections by two observers. Cellular infiltrate in both tumor stroma and tumor parenchyma was assessed by counting lymphoreticular cells in each tissue compartment in a minimum of ten microscopic high power fields and averaged. 

### 2.4. Statistical Analysis

Statistical correlations were calculated using the BMDP (biomedical data processing) package from UCLA (University of California, Los Angeles, 1985 version). The parameters included in the study are listed in Table 2. First, tumors were divided in two groups according to the mean value of LCs per 10 high-power fields (HPFs), which was found to be 7.32: tumors with less than 7 LCs/HPF and tumors presenting more than 7 LCs/HPF. A chi-squared test and, whenever appropriate, Fisher's exact test were applied for analysis of contingency tables. The analysis of the variance test was used for continuous variables (natural log transformed when necessary). Survival was estimated by the Kaplan and Meier method, and survival curves were compared with the log rank test. Cox's proportional hazards survival analysis was used to determine the relative risk in multivariate analysis.

## 3. Results

**Figure 1:**
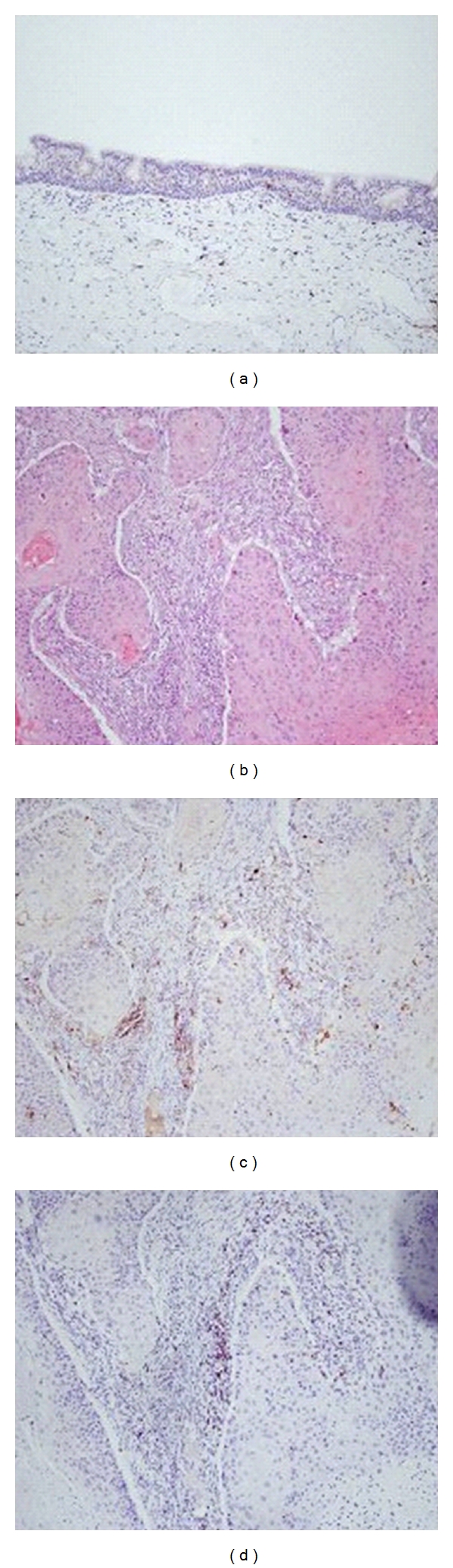
Immunostaining of Langerhans cells (CD1) in a larynx normal tissue (a) and paraffin sections of a epidermoid carcinoma of larynx stained with H&E (b) and immunostained for Langerhans cells (CD1) (c) and cytotoxic T lymphocytes (CD8) (d).

LCs were present in most of the primary tumors investigated (44 out of 50 cases) ranging from 0.6 to 18.3 (mean 7.32, standard deviation 4.5). The presence of LCs was detected in all mucosa samples distant to tumor from the patients (Figure 1) which were found to be polystratified epithelia, more often keratinized. The number of LCs varied according to the thickness of the mucosal layer and the degree of keratinization. They were more frequently found in polystratified and keratinized epithelia. LCs were also found in lymph node metastases (6 out of 10) and recurrences (2 out of 3). 

When analyzing the relationship between number of LCs and the parameters studied, Langerhans cells were increased in patients with evident infiltration of lymphocytes, mainly cytotoxic T lymphocytes (Figure 1, Table 1). Thus, an increase in LCs was significantly associated with an increase in number of leukocytes, cytotoxic T lymphocytes and B-cells, but a decrease in the number of macrophages and in the ratio of CD4/CD8, which emphasize the role of LCs as antigen-presenting cells. 

On the other hand, we were not able to find any statistical association between clinical-pathological parameters, prognosis and number of LCs (Tables 2 and 3).

## 4. Discussion

LCs can be readily identified immunocytochemically in a large series of human laryngeal tumors even though they are rare in normal larynx [14]. Although LCs were originally identified by silver staining methods, the advent of immunocytochemical techniques and Moabs has considerably aided their recognition. Antibodies against a variety of different antigens have been successful in demonstrating LCs. Those against CD1 antigen, such as OKT6 used in this study, are generally recognized as sensitive in distinguishing LCs from other cell types in the skin and nonlymphoid tumors [15, 16]. In particular, they are superior to others like anti-HLA-DR and anti-S-100 protein, both of which recognize a number of other cell types such as macrophages or melanocytes which may cause confusion with LCs. Since the only other cell labelled by anti-CD1 antibodies is the immature thymocyte, there is a little chance of any confusion of LCs with other cell types using these reagents. 

In the current series no correlation was found between number of LCs and parameters of prognostic value. In breast and uterine carcinomas an association between high number of LCs and low-stage tumors has been reported [17, 18]. However, similar to our results, in previous series of larynx carcinomas this association was not found [7]. This suggests a different role (immunorecruitment) for these peculiar cells (see Table 1), more than a first line of defence.

Concerning prognosis, it is tempting to speculate that tumors presenting an increase number of Langerhans cells would be prone to pursue a more favourable clinical course. In fact, in gastric carcinoma [3], thyroid carcinoma [4], and early-stage lung carcinoma [19], numbers of LCs are correlated with a better prognosis. However, in larynx carcinoma positive and negative findings have been reported [8, 9]. Our series has a long followup and we were not able to find a relationship with survival. Probably the different results in the literature could arise from the different methods of identification employed, as not all the studies used Moabs against CD1 antigen which is considered to be highly specific for LCs. We should also consider that cancer survival is multifactorial and unlikely to be determined by a single alteration. Therefore, the LC is not a reliable marker to determine prognosis of the patients with laryngeal squamous cell carcinomas in the clinical practice. However, it is conceivable that the presence of LCs could enhance the host immune response against the tumor through the ability to recruit cytotoxic T lymphocytes.

## 5. Conclusion

The number of LCs is not a useful tool to determine the prognosis in laryngeal cancer patients but the direct relationship between LCs and immunomodulatory cells may influence the lysis of laryngeal cancer cells.

## Figures and Tables

**Table 1:** Relationship between LCs and subpopulations in tumor-infiltrating leukocytes in squamous cell carcinoma of the larynx.

Tumors with ≤7 LC/HPF versus tumors with >7 LC/HPF*			
Means values in each group of tumors			
Parameter**	≤7 LC/HPF	>7 LC/HPF	Significance

Leucocytes (CD45)	60.5	92.4	*P* = 0.0037
Macrophages (MO1)	19.07	14.48	*P* = 0.0291
B cells (IOM-1)	1.69	3.85	*P* = 0.0234
T cells (CD3)	31.2	51.6	*P* = 0.0436
Cytotoxic/suppressor T cells (CD8)	14.4	25.7	*P* = 0.0435
T cell/macrophage ratio (CD3/CD11b)	1.97	3.79	*P* = 0.0085
CD4/CD8	2	1.2	*P* = 0.0075

*Average counting 10 high-power fields. **Mean values for number of TIL or ratios.

**Table 2:** Relationship between LCs and clinicopathologic parameters in squamous cell carcinoma of the larynx.

Tumors with ≤7 LC/HPF versus tumors with >7 LC/HPF*			
Parameter	≤7 LC/HPF	>7 LC/HPF	Significance

Site			
Supraglottic	14	13	N.S
Other sites	9	14	
T stage			
I + II + III	16	22	N.S
IV	7	5	
Neck metastasis			
Yes	7	9	N.S
No	16	18	
Differentiation			
well to moderately differentiated	18	22	N.S
Poorly differentiated	6	4	
Jakobsson**	19.4 ± 1.1	18.1 ± 0.9	N.S
Glanz**	5.5 ± 0.4	5.6 ± 0.4	N.S

*Average counting 10 high-power fields. **Mean value + mean standard error.

**Table 3:** Variables associated with overall survival.

Variable	O.R (Univariate) (95% CI)	O.R (multivariate) (95% CI)
T stage		
(IV versus I–III)	4.5 (1.5–14.3)	5.1 (1.5–17.2)
Neck metastasis		
Yes versus No	9.5 (2.5–35.5)	8.4 (2.2–31.9)
Jakobsson		
>16 versus ≤16	1.9 (1.2–2.9)	—
Glanz		
>5 versus ≤5	5 (1.1–23)	—
LCs		
>7 versus ≤7	1.5 (0.4–5.5) N.S	—

O.R: odds ratio, CI: coefficient interval, N.S: nonsignificant.
